# Scan, dwell, decide: Strategies for detecting abnormalities in diabetic retinopathy

**DOI:** 10.1371/journal.pone.0207086

**Published:** 2018-11-16

**Authors:** Samrudhdhi B. Rangrej, Jayanthi Sivaswamy, Priyanka Srivastava

**Affiliations:** 1 Center for Visual Information Technology, International Institute of Technology, Hyderabad, India; 2 Cognitive Science Lab, International Institute of Information Technology, Hyderabad, India; University of Louisville, UNITED STATES

## Abstract

Diabetic retinopathy (DR) is a disease which is widely diagnosed using (colour fundus) images. Efficiency and accuracy are critical in diagnosing DR as lack of timely intervention can lead to irreversible visual impairment. In this paper, we examine strategies for scrutinizing images which affect diagnostic performance of medical practitioners via an eye-tracking study. A total of 56 subjects with 0 to 18 years of experience participated in the study. Every subject was asked to detect DR from 40 images. The findings indicate that practitioners use mainly two types of strategies characterized by either higher dwell duration or longer track length. The main findings of the study are that higher dwell-based strategy led to higher average accuracy (> 85%) in diagnosis, irrespective of the expertise of practitioner; whereas, the average obtained accuracy with a long-track length-based strategy was dependent on the expertise of the practitioner. In the second part of the paper, we use the experimental findings to recommend a scanning strategy for fast and accurate diagnosis of DR that can be potentially used by image readers. This is derived by combining the eye-tracking gaze maps of medical experts in a novel manner based on a set of rules. This strategy requires scrutiny of images in a manner which is consistent with spatial preferences found in human perception in general and in the domain of fundus images in particular. The Levenshtein distance-based assessment of gaze patterns also establish the effectiveness of the derived scanning pattern and is thus recommended for image readers.

## Introduction

Diabetic Retinopathy(DR) is a disease that affects the inner wall of the eye, namely, the retina. Individuals in the age group of 20 to 74 years and with Type-I and Type-II diabetes have a higher risk of developing DR [1]. DR is a major cause of visual impairments and in some cases leads to permanent blindness. Early detection of DR and its treatment can prevent vision loss.

Just as mammograms form the frontline of breast cancer screening, images of the retina (commonly known as Fundus images) have been explored and recommended for DR screening. These images are scrutinized by experts or trained readers in reading centres (e.g. Doheny Image Reading Center (DIRC) [2]) to give preliminary diagnosis. Automated reading by employing computer-aided diagnostic (CAD) algorithms has also been explored in the last decade for scaling up the screening process.

Understanding the strategies used by various retinal experts is crucial to train readers and to develop *human-like* CAD tools. Training of both human and CAD tool requires large amount of images with ground truth from retina experts. Tedium of marking and the priority of patient care (over marking), impedes the collection of ground truth. Hence, recently, efforts have been made to explore crowd-sourcing to acquire abundant manual annotation [3–6]. Individuals with various levels of skill participated in these trials. The relationship between accuracy of collected annotation and skill-level was observed to be rather complex [4]. This indicates that accuracy of diagnosis depends on multiple aspects opposed to just one, viz expertise. Understanding of these aspects is long overdue. In this paper, we aim to discover such aspects specific to the fundus image-based DR diagnosis process and examine their role along with expertise. Specifically, we aim to evaluate the best practices and strategies used for DR diagnosis.

*Eye-tracking* is a popular technique which is used extensively to investigate visual perception in a range of tasks. Eye tracking in a screening task enables evaluation of the conscious and subconscious aspects of perception. The conscious aspect pertains to what and where to look in a given image. This involves domain knowledge, which directs the search for specific abnormalities in the specific location(s) while ignoring the normal anatomical structures. Subconscious aspect is understood as how to look. This involves the gaze pattern, dwelling and time delay which are difficult to teach or learn. Given the importance of evaluation of eye-movement in the diagnostic process, eye-tracking studies have been of interest and reports on 2D and 3D images can be found in the literature starting from X-ray [7, 8], to CT images [9–11], microscopy [12–14] and mammography [15, 16]. However, screening for DR by detecting lesions in fundus images has, to the best of our knowledge, not been investigated.

**Fig 1:**
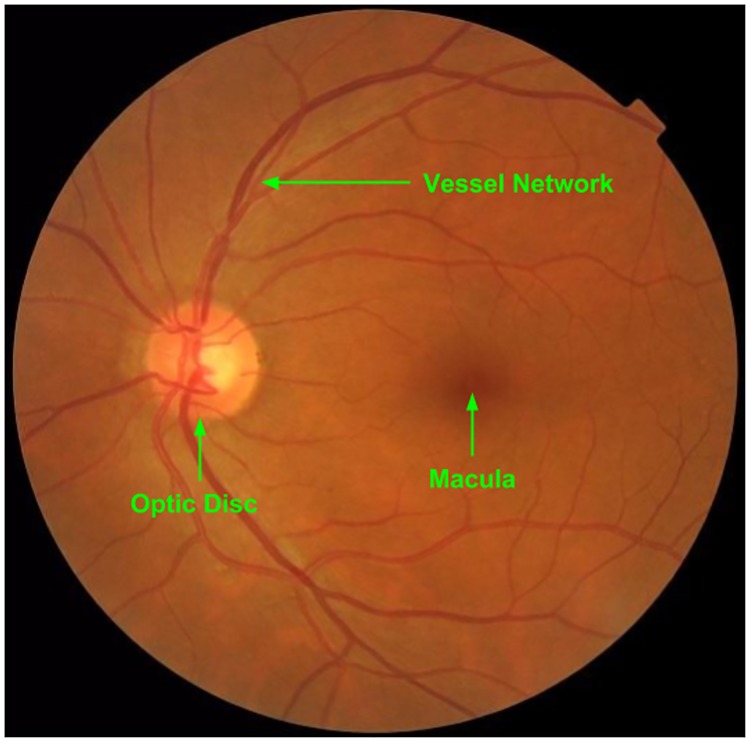
Retinal surface with three major structures.

The fundus image is a projection of the retinal surface capturing three major structures: Optic Disc, vessel network, and macula/fovea (see Fig 1). The first two are irrelevant for early detection of DR and is ignored by practitioners with a short training. The macula is responsible for color vision with high acuity and hence is of specific clinical interest. The locations of lesions relative to macula determine the severity of the disease and this knowledge is gained only from medical training. Given the vital role of knowledge in identifying the possible locations and their relative diagnostic value, it becomes necessary to evaluate the scanning strategies as a function of knowledge in DR.

In this paper, we describe an eye tracking study, conducted to understand strategies used by subject with varying degree of expertise in DR screening. The study was restricted to the detection of two types of lesions which occur in the early phase of DR, namely, hard exudates and hemmorhages, which occur due to leakages of lipid and blood, respectively. Finally, the results of the eye tracking study are used to extract a scanning pattern that can be recommended for image readers for efficient and accurate DR screening.

## Eye tracking experiment

### Stimuli images

A dataset of 145 abnormal and 50 normal fundus images was obtained from local eye hospital. A subset of 40 images with approximately equal number of normal and abnormal cases was selected by senior retina consultant from this dataset. This subset contains images with various level of diagnostic difficulties.

### Participants

In order to maintain diversity among the participants, retina experts were invited from 6 different eye hospitals. A total of 44 retina experts accepted the invitation and participated in the study. Retina experts were classified into 3 categories, namely, consultants, fellows and residents/optometrists (see Table 1). 12 engineering students from our home institute were also included to fulfill the role of novices. They were given a short training with 10 example fundus images.

**Table 1:** Description of participants.

Level of expertise	Number of participants	Range of experience (years)
Consultant	13	7-18
Fellow	17	3-7
Resident/Optometrist	14	1-12
Novice	12	0

Total number of image and number of images with hard exudate/hemorrhage are shown.

### Material

**Fig 2:**
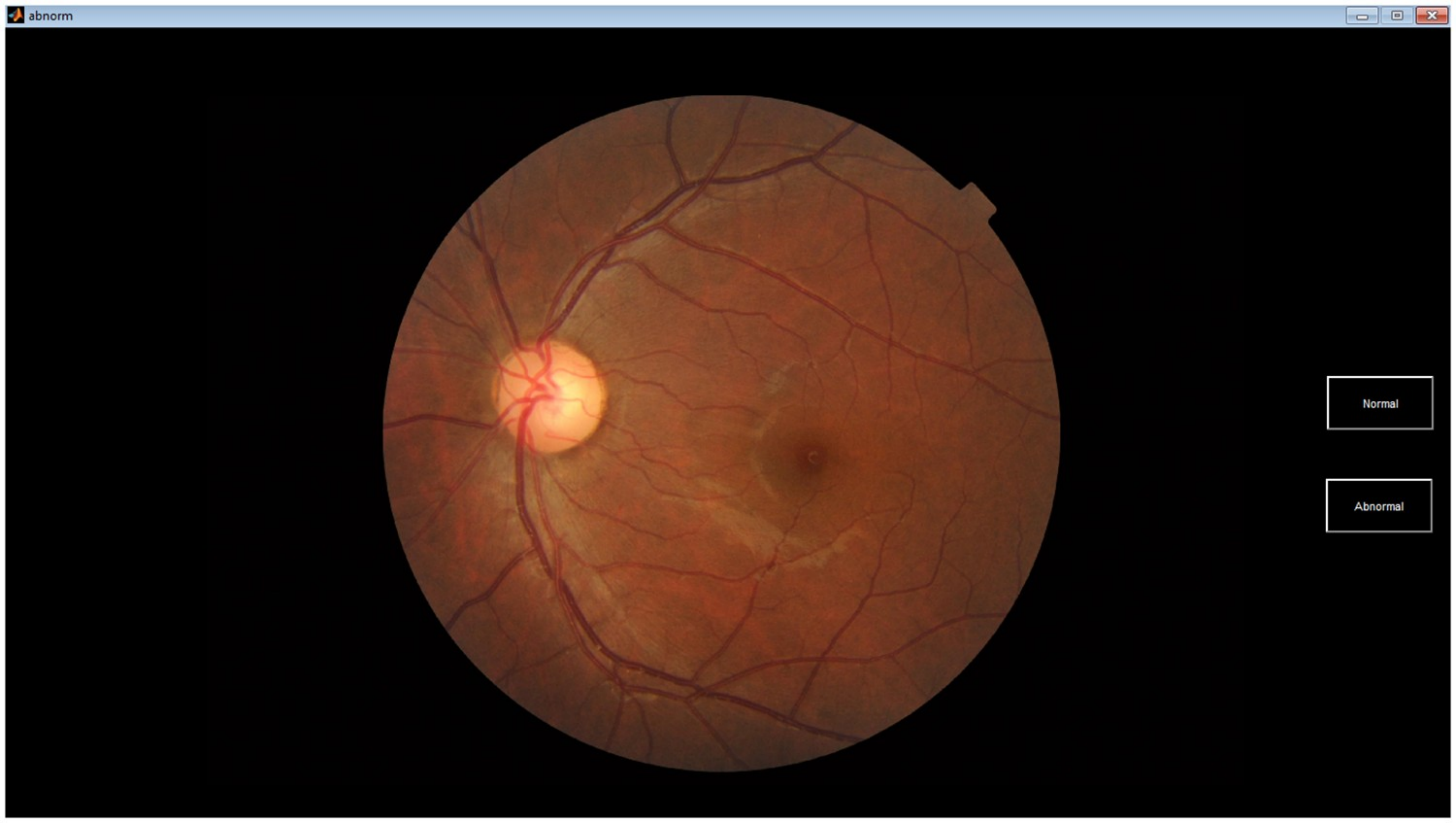
Example display from the software tool developed for the experiment.

Experiments were carried out in a dark room with constant environment condition. Tobii X2-30 eye tracker (sampling rate 30Hz) with a 15.6” display screen were used. Tobii X2-30 eye-tracker allows free head movement of 20″ × 14″ (Width × Height) at the distance of 70m (distance may vary from person to person) [17]. This range is compatible with the size of used display. Stimuli (images) were down-scaled to fit the display size. A software tool was developed in MATLAB 8.2 (see Fig 2) for use in the study to help participants view images and give their decisions. A small tutorial was also given to all participants to ensure easy usage of the software.

### Experiment design

**Fig 3:**
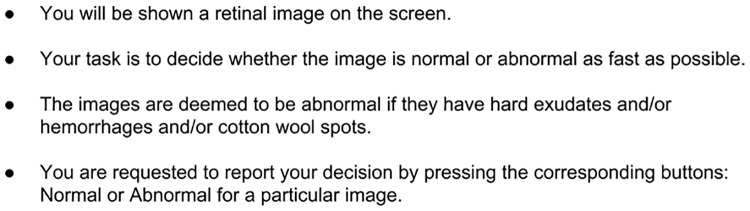
Instructions given to participants.

The study was approved by an ‘Institute Ethics Committee of International Institute of Information Technology, Hyderabad, India’ constituted for this purpose. The committee approved the protocol of taking informed consent: signing an e-form by the subjects. The experiment proceeded only after this form was signed. The instruction given to all participants is shown in Fig 3. A 5-point calibration was performed at an interval of 5 images. The response time, response category (normal vs abnormal decision) and eye position (*x*, *y*) with timestamp were stored for each trial. The session lasted for approximately 20 minutes. Eye positions were classified into fixation, saccade, and glissade using the adaptive algorithm proposed in [18]. Glissades are corrective eye-movement when eye undershoot/overshoot the intended target. It is widely believed that glissades are just remedial movements and they do not serve any useful purpose [19]. Hence, Eye positions classified as glissade were removed from the data and the remaining data were registered to actual image size (in pixels).

## Definitions

We begin with definitions of some quantities used for analysis of eye tracking data. All quantities were calculated for the eye tracking data registered to original image size in pixel units.

### Total track length

**Fig 4:**
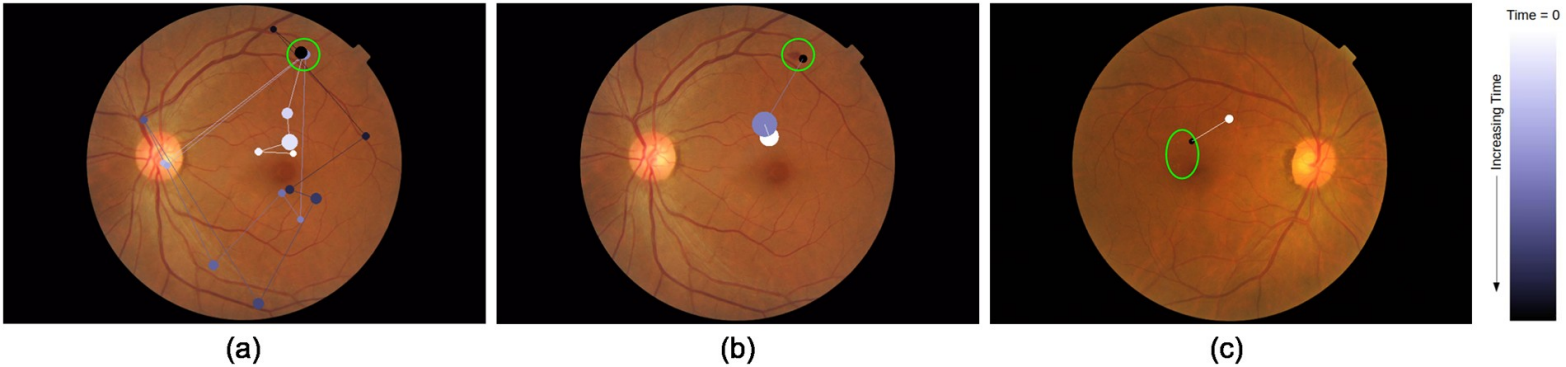
Total track length for sample images. (a)-(b) Gaze-tracks for two different subjects for an image with a lesion (marked with green circles) in the periphery. One subject takes longer route than another. (b)-(c) Gaze-track for an image with peripheral and more centrally located lesion. Image with a lesion at periphery(center) requires longer(shorter) total track length.

The Euclidean distance between two consecutive fixations is defined as track length in *pixel* unit. The sum of all the track lengths for an image *j* for an *i*^*th*^ participant is defined as the total track length *l*_*ij*_. The value of *l*_*ij*_ depends on the subjective behavior of the participant as well as the location of the lesion in the image. Fig 4 illustrates these points. Fig 4(a) and 4(b) show tracks for 2 subjects on one image. It is evident that one subject takes a very long route whereas the other takes a more direct route to the lesion location indicated by a green circle. Assuming that the visual search starts from the center, an image with lesion(s) in the peripheral region will have larger *l*_*i*,*j*_ than an image with lesion(s) at the center. This is evident from Fig 4(b) and 4(c).

### Standardized track length

The effect of the location of lesions on *l*_*ij*_ has to be nullified in order to capture inter-subject variability. The nullification is done by dividing *l*_*ij*_ by sum of total track length of all the participants for *j*^*th*^ image. The standardized track length(*L*) is defined as follows.

$$L_{ij}=\frac{l_{ij}}{\sum_{i}l_{ij}}$$ (1)

From the definition, it follows that Standardized track length is a unitless quantity whose value lies between 0 and 1.

### Total dwell duration

**Fig 5:**
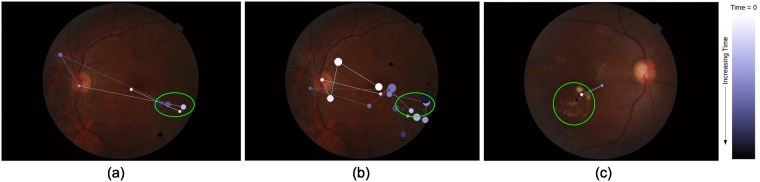
Comparison of dwell duration. Dwell duration is proportional to the radius of the disc representing fixation. Lesion locations are marked with green ovals. (a)-(b) Gaze-track for two different subjects for an image. Very different dwell pattern shows total dwell duration depends on subjective behavior. (b)-(c) Gaze-track for two different stimuli images. Gaze-track for image with subtle(prominent) lesion has more(less) total dwell duration.

The time spent on each fixation is defined as dwell duration which is in *micro-seconds*. The sum of dwell durations for each fixation (including revisits) is considered as total dwell duration. Total dwell duration of *i*^*th*^ participant for *j*^*th*^ image is denoted as *d*_*ij*_ which also depends on the subjective behavior of the participant and the conspicuity of lesions in the image. Fig 5 illustrates this point. Fig 5(a) and 5(b) shows the dwell pattern of two different subjects where one subject has fewer fixations than the other and hence lower total dwell duration. Fig 5(b) and 5(c) on the other hand illustrates the dependency of *d* on conspicuity. Here, an image with subtle lesions is seen to lead to longer dwell duration per fixation in a subject than an image with prominent lesions.

### Standardized dwell duration

The effect of conspicuity of lesion on *d*_*ij*_ has to be nullified in order to capture inter-subject variability. This is done by dividing *d*_*ij*_ by the sum of total dwell duration of all subjects. The resultant dwell duration is termed as standardized dwell duration(*D*) and is defined as follows.

$$D_{ij}=\frac{d_{ij}}{\sum_{i}d_{ij}}$$ (2)

Standardized dwell duration is also an unitless quantity taking values between 0 and 1.

### Coefficient of scanning

This is defined as the ratio of standard dwell duration and standard track length.

$$CS_{ij}=\frac{D_{ij}}{L_{ij}}$$ (3)

*CS* is positive valued and theoretically, *CS* = ∞ when *L* is zero. However, this is possible only if there is just one fixation for an image and this is practically nearly impossible and hence, *CS* can assume any large (< ∞) positive value. Coefficient of scanning represents the balance between track length and dwell duration and thus can provide insights into scanning strategies of subject underlying the Normal/Abnormal decision task.

## Dwelling vs Tracing

**Fig 6:**
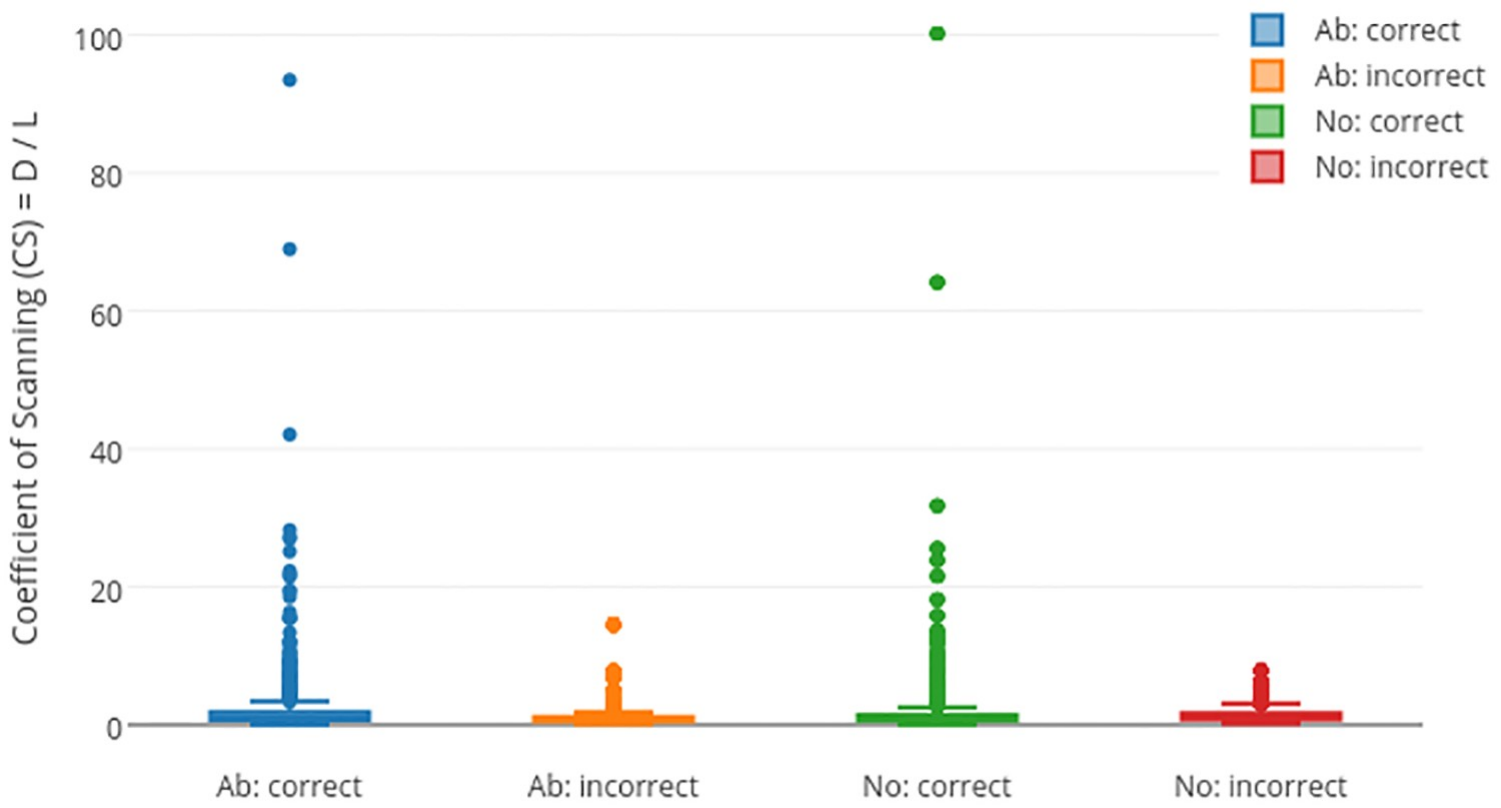
*CS* values for 2240 responses.

Responses of the 56 participants for 40 images result in a total of 2240 responses. These were divided into 4 categories: (i) correct response for abnormal case (True Positive) (ii) incorrect response for abnormal case (False Negative) (iii) correct response for normal case (True Negative) and (iv) incorrect response for normal case (False Positive). *CS* was computed for each of these categories and the results are shown in Table 2 and Fig 6. *CS* is seen to be significantly higher for True responses than False responses for both normal and abnormal cases. The average *CS* value for the four groups is 1.5.

**Table 2:** Average coefficient of scanning for various image category and response pair.

Image category: Response	*CS*	p-value
Abnormal: Correct	2.1 ± 5.0	8.97 × 10^−7^
Abnormal: Incorrect	1.2 ± 1.6	
Normal: Correct	1.6 ± 4.5	4.07 × 10^−4^
Normal: Incorrect	1.4 ± 1.5	

Total number of image and number of images with hard exudate/hemorrhage are shown.

This average *CS* = 1.5 value was used to analyse the responses. Responses with *CS* ≤ 1.5 implies *D* ≤ 1.5*L*, i.e. dwelling is less but the gaze-track is long. Hence, we call this scanning strategy as *Tracing*. On the other hand, responses with *CS* > 1.5 has *D* > 1.5*L*, i.e. gaze-track is short but dwelling is more. We call this scanning strategy as *dwelling*.

### Performance analysis of *Dwelling* and *Tracing* strategies

**Fig 7:**
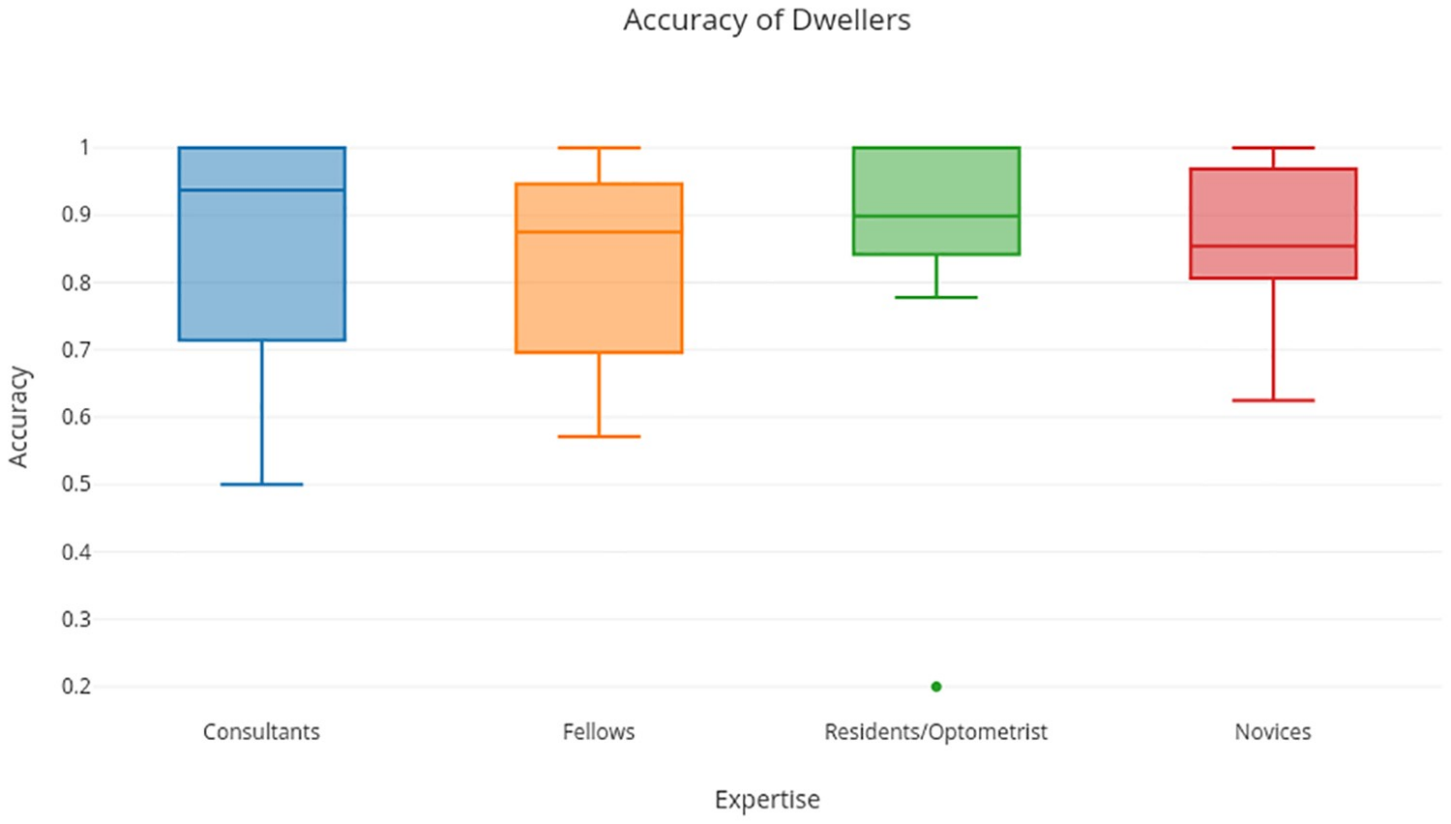
Accuracy achieved with *dwelling* strategy.

Table 2 indicates an interesting trend in that corresct responses go with a *Dwelling* strategy as *CS* > 1.5 for such responses (first and third rows). The accuracy achieved by various subject groups with *dwelling* strategy is shown in Fig 7. Most of the participants have accuracy > 85%. Achieved accuracy is thus high regardless of the expertise level of the participant (p = 0.5730).

**Fig 8:**
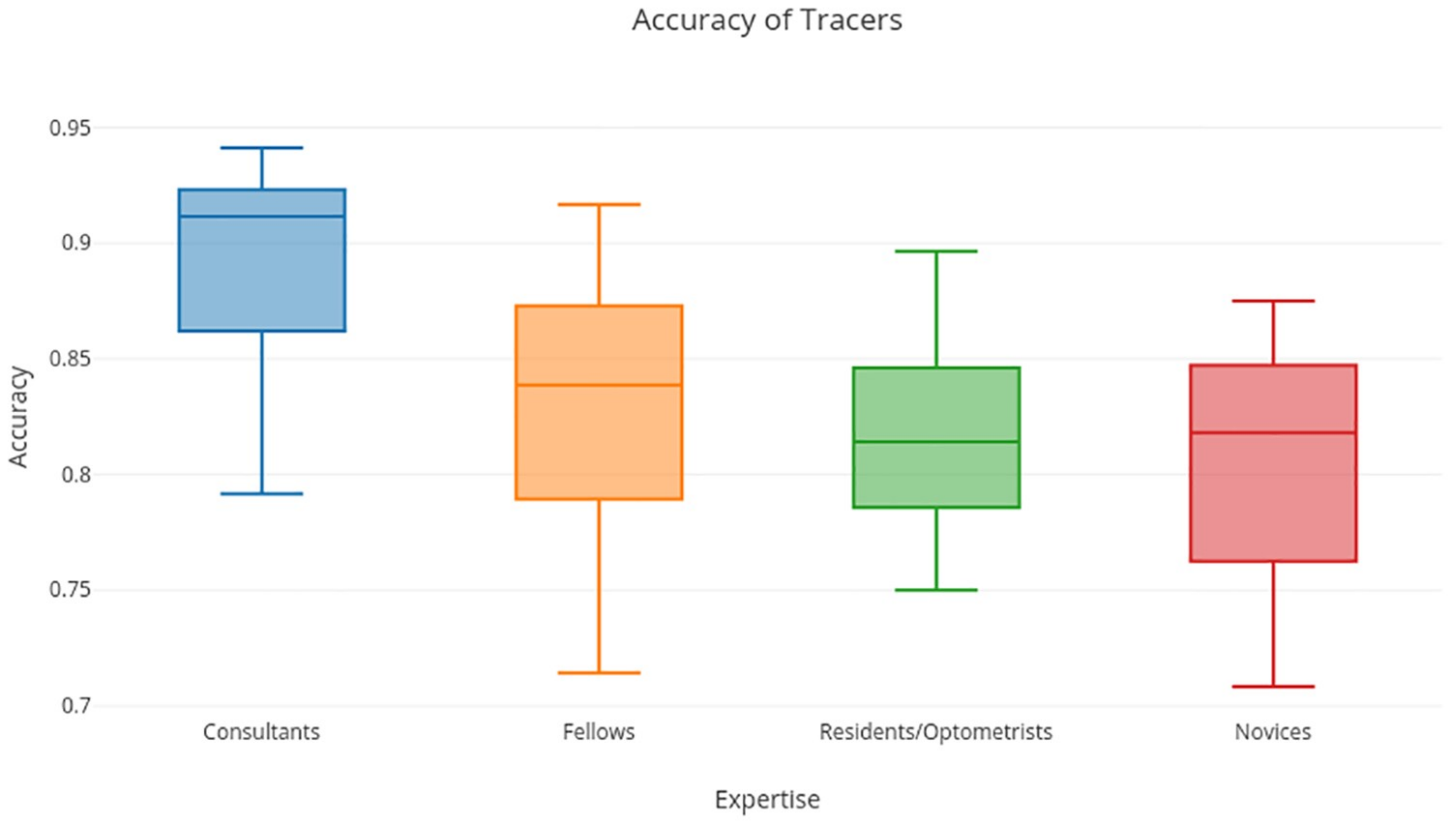
Accuracy achieved with *tracing* strategy.

*Tracing* strategy, on the other hand, leads to a lower accuracy and analysis also shows that expertise affects the performance with this strategy. Specifically, accuracy decreases with decreasing levels of expertise (p-value = 0.0057) (see Fig 8). In order to understand this trend, further analysis of the gaze pattern was done.

**Fig 9:**
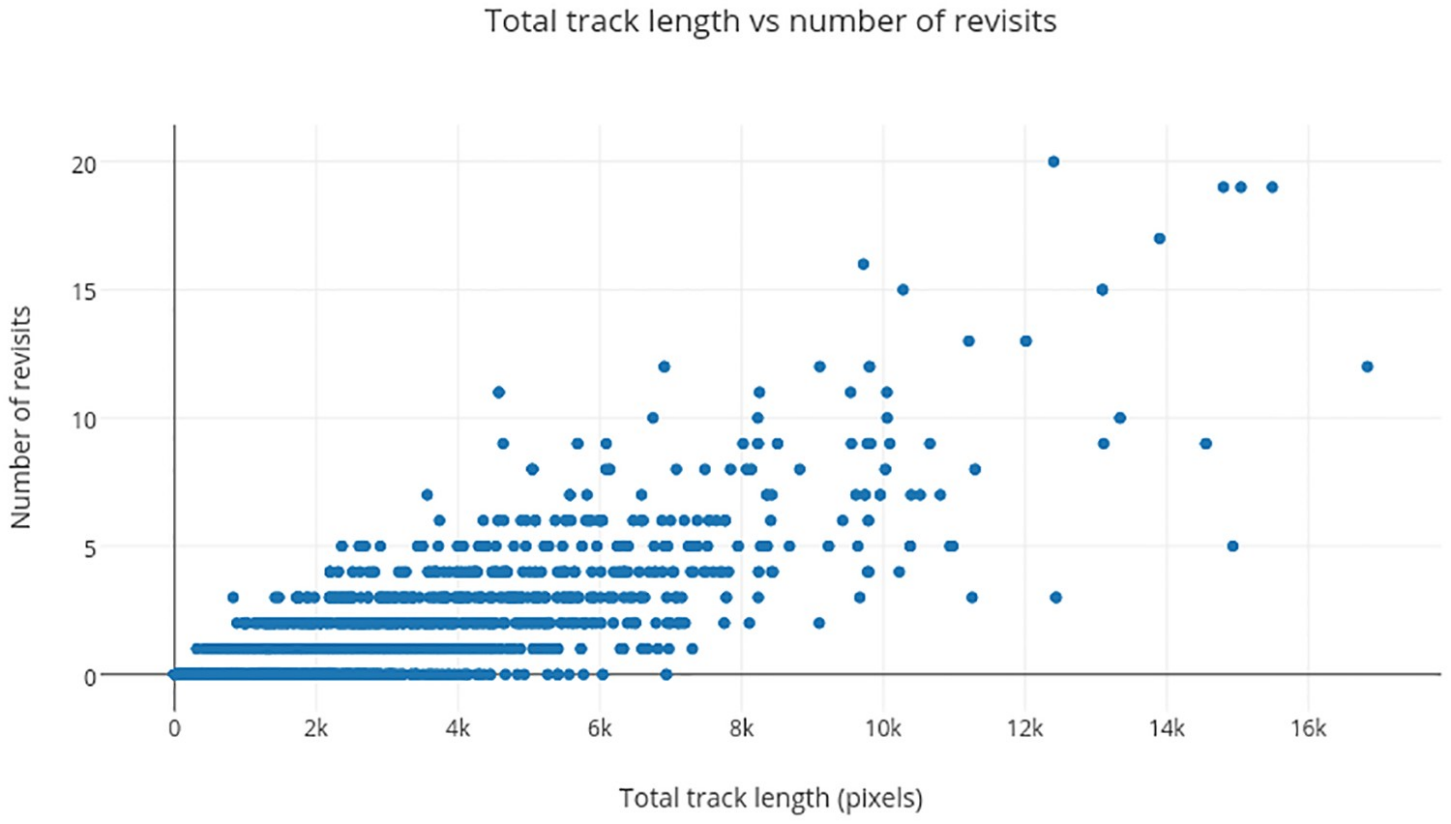
Correlation between track length and revisits.

*Tracing* is characterized by higher standardized track length. Ideally, longer track length should be a result of greater spatial coverage. However, longer track length can also be due to revisits to regions scrutinized earlier. Therefore, the correlation coefficient between track length and number of revisits was computed. This was found to be 0.8. This implies, the number of revisits increases with track length (see Fig 9). This can also be seen from the sample case shown in Fig 4(a) where a subject had longer gaze-track due to revisit. Also, based on performance analysis of the *tracing* strategy, we can conclude that revisits appears to be beneficial for subjects to make a correct decision only if they have more expertise.

*Dwelling* is also faster than *tracing* as the average response times with *dwelling* and *tracing* are 4.6s and 5.4s, respectively. Thus, *dwelling* appears to be an efficient strategy for accurate and fast decision, regardless of a subject’s expertise level.

## Discussion

The aim of this study was to understand the following. (a) Examine the strategies used by various experts in diagnosing DR by scrutinising a fundus image. (b) Understand role of knowledge in diagnosing DR from fundus images.

The current eye-tracking results indicate that readers use mainly two kinds of strategies while reviewing fundus images, namely, tracing and dwelling. Subjects were found to choose either of these strategies, independent of their previous experience (p-value >> 0.05). Expertise appeared to be beneficial to subjects in making accurate decisions with tracing as experts showed significantly higher accuracy with DR detection compared to semi-experts and novices, while tracing. Since tracing entails low dwell time for a particular region, it leads a reader to review the region over multiple revisits with the net effect of a long gaze-track and a slow response. That revisits favored experts more than semi-experts and novices indicates the importance of prior experience or knowledge in a given domain. An informal conversation with Retina experts also supports the current finding that tracing benefits experts more than semi-experts and novices. It can be argued that revisiting enables readers to gather more evidence to support decision making but might not help semi-experts or novices who lack the ability to gather and assess the information in the region. However, the scope of generalization could be limited to the DR as of now.

The dwelling strategy, in contrast, involves longer dwell time for a region which is better suited to gather adequate information for decision making. This is affirmed by the fact that participants employing a dwelling strategy have higher accuracy and faster response time. The long scrutiny enables full attention to a region which should be beneficial to a participant in overcoming any lack of experience and expertise, which might be the reason for the high accuracy of decision achieved by all the groups regardless of their expertise. As dwelling requires scrutinizing regions for a longer time, a decision is possible with less number of reviews and consequently, the total track length and response time is low. This makes dwelling an efficient and faster strategy.

The current results have some similarity with the previous finding [20] on 3D Computed Tomography (CT) images, which identified a drilling (stepping across slices to decide about a location) versus scanning (within a slice) strategy. The findings indicated higher detection accuracy with a drilling (as opposed to scanning) strategy. In the absence of additional slices (3D information), drilling might be akin to dwelling. Our result suggests that dwelling has an advantage over tracing strategy. This trend is particularly evident in the case of novices and semiexperts, whose detection accuracy drops to approximately 80%, during tracing strategy. Although dwelling seems to be a better strategy, tracing was found to be used more by all subject groups as 75% of the responses were made using tracing and remaining with dwelling. Also, each subject was found to use different strategy for different images (tracing for 75% of images and dwelling for 25% images). Furthermore, the choice of strategy for particular image did not depend on the challenging (prominent versus subtle lesions) nature of detection (p > > 0.05). It is noteworthy that the average accuracy of detection across subject groups, irrespective of the strategy used, was above 80%. The specific values were as follows: 89.50%, 83.24%, 83.57%, 81.87% for the consultants, fellows, residents/optometrists and novices, respectively. The difference between these values is significant (p = 5 × 10^−4^) suggesting the role of expertise is important. The high average accuracy achieved by novices, which is due to the relatively easy nature of the detection task, is also consistent with the high level of agreement reported between the crowd-sourced image annotations and expert marked ground truth for DR [3–5].

So far the analysis of the gaze-maps has not incorporated the knowledge of the underlying anatomy of retina which is characterised by a central region (macula) which is critical for vision. The ETDRS [21] guidelines for DR detection prescribe a set of 9 zones for review during diagnosis and DR staging is done based on the health of these zones. Next, we analyze the gaze-maps using these zones. We examine the order in which retinal zones are scrutinized to identify the variation, if any, in the zone-wise gaze pattern across expertise.

## Scanning pattern analysis

**Fig 10:**
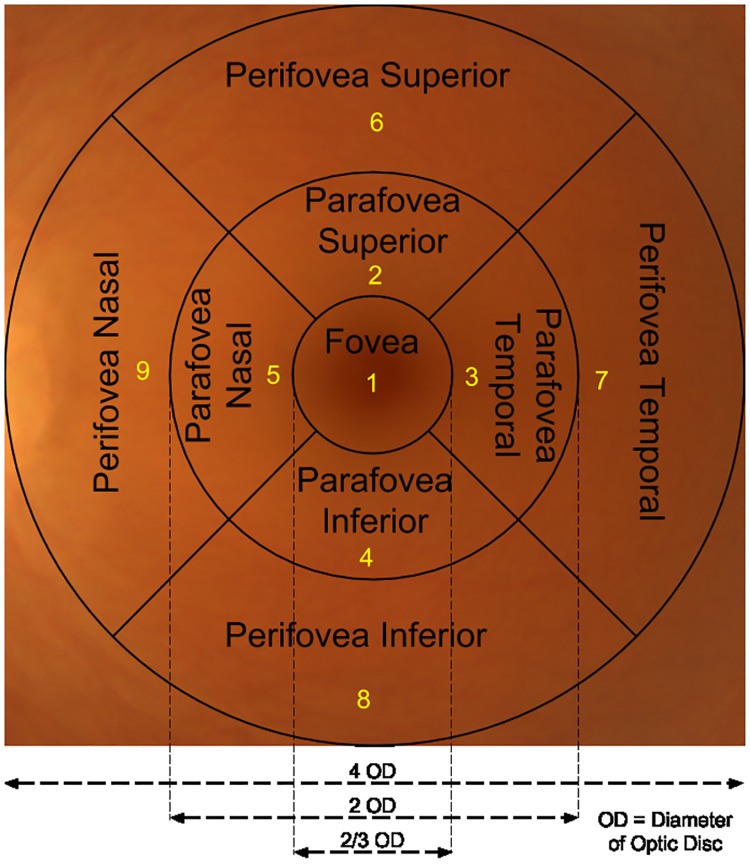
Retinal zones recommended by ETDRS.

The 9 zones of a fundus image recommended for review during diagnosis are shown in Fig 10. In this section, eye movement patterns are analyzed in terms of the gaze transitions between these zones and zone-wise dwelling. As the aim is to understand scanning patterns for accurate diagnosis, responses with only correct decision are considered.

### Transition pattern

**Fig 11:**
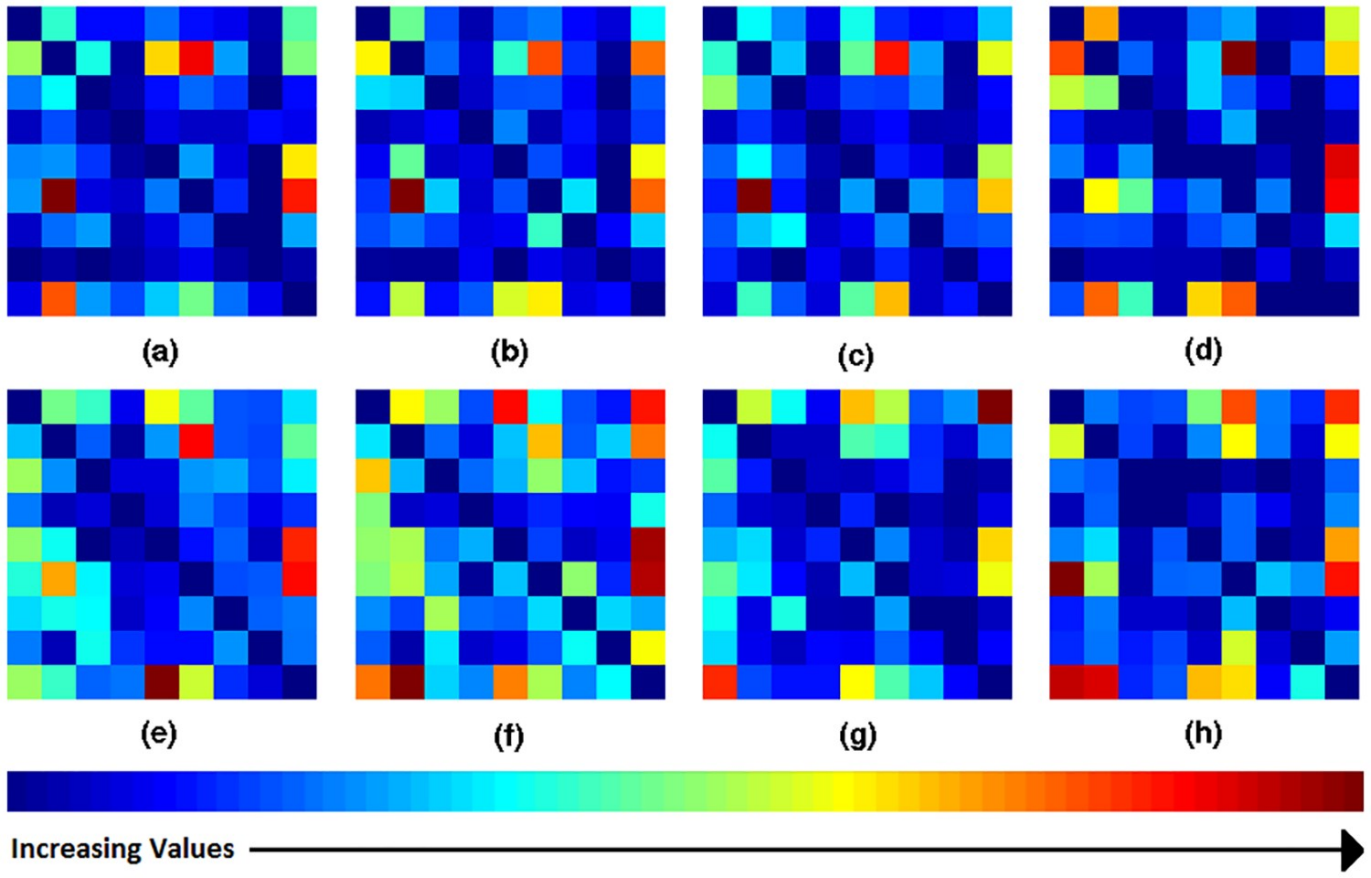
Average transition matrices for different expertise-groups. Top/bottom row: abnormal/normal cases. Left to right: consultants, fellows, residents/optometrists, novices. Each transition matrix is scaled and color-coded separately.

Transition is defined as the movement of eye-gaze from one zone to another. Each retinal zone was assigned a number as shown in Fig 10. A matrix with the number of transitions from zone *j* to zone *i* as entries was computed and termed as the transition matrix (*TM*) [22]. The transition matrix for abnormal and normal cases were analyzed separately. The computed average transition matrix for four expertise-groups is shown in Fig 11. This matrix was not observed to be significantly different across expertise-groups with medical training (abnormal: *p* = 0.0804, normal: *p* = 0.2712). Whereas, the average *TM* for novices is significantly different from the other expertise-groups (abnormal: *p* = 0.0022, normal: *p* = 0.0041).

### Dwell map

A dwell map showing the average time spent on dwelling, in a zone, was also constructed. This was done by first summing the dwell duration for fixations falling in a particular zone. This sum is defined to be the zone-wise total dwell duration. Total dwell duration of the *k*^*th*^ zone for the *i*^*th*^ participant and *j*^*th*^ image is denoted by *d*_*ijk*_. Next, an average zone-wise total dwell duration for expertise-group *E* was computed as follows.

$$d_{k}\left(E\right)=\frac{1}{ij}\sum_{i,j}d_{i,j,k};\;i\in E_{j}$$ (4)

**Fig 12:**
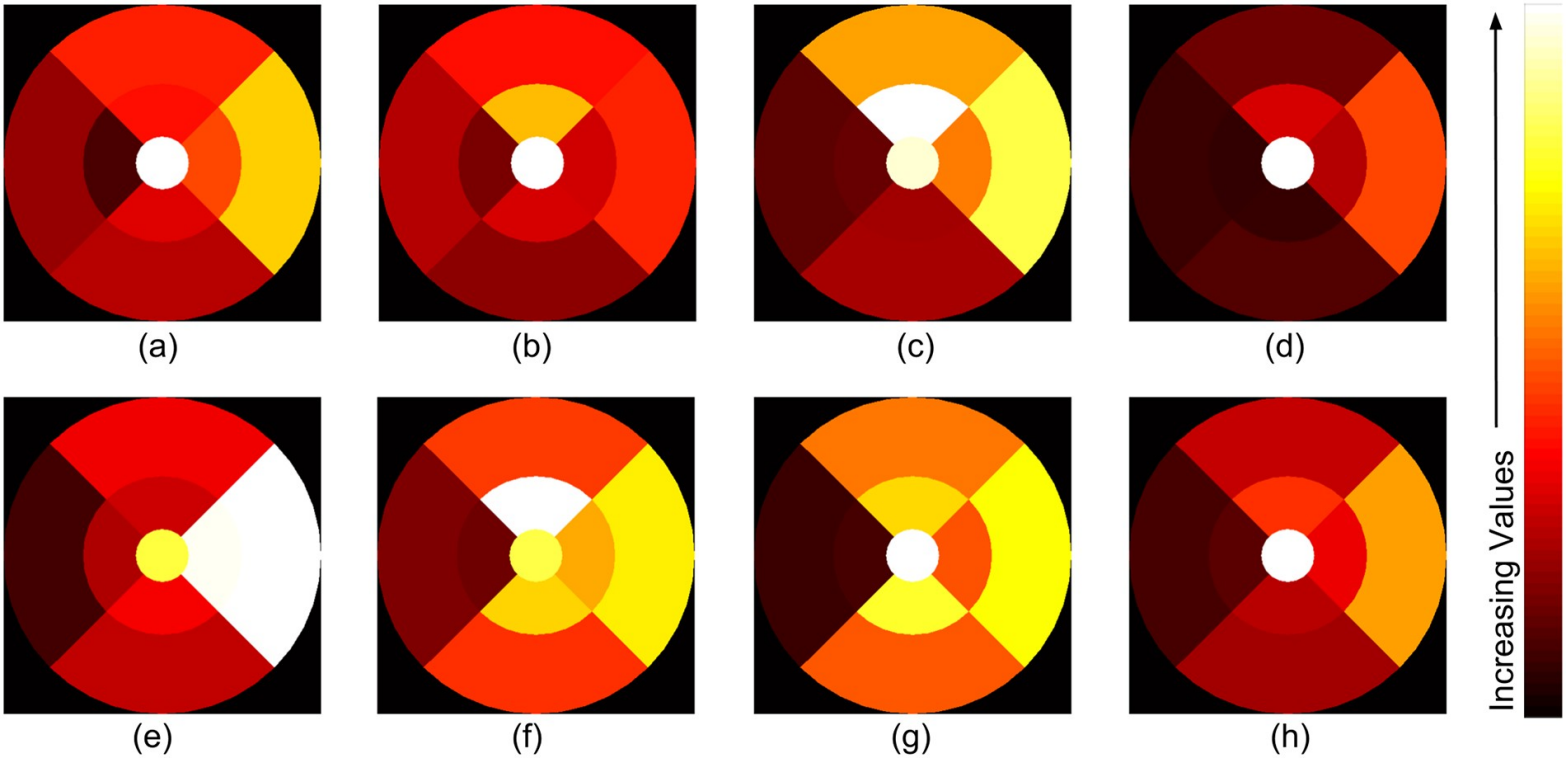
Average dwell maps for different expertise-groups. Top/bottom row: abnormal/normal cases. Left to right: consultants, fellows, residents/optometrists, novices. Each dwell map is scaled and color-coded separately.

Here, *E*_*j*_ is the set of participants who gave a *correct* response for the *j*^*th*^ image and belong to expertise-group *E*. Average dwell maps of various subject groups for abnormal and normal images are shown in Fig 12. Difference between *d*_*k*_(*E*) for the four expertise-groups was not significant (abnormal: p = 0.5726, normal: p = 0.9375).

The results of zone-wise analysis indicate that time spent on various zones by different expertise group is similar but the order in which these zones are reviewed are not. The order of review changes significantly with medical training (expertise), but it does not depend on experience of medical experts.

## Recommendation

In order to *train* a human reader or a human-like CAD tool to analyse fundus images in a manner similar to a medical expert, an image reading guideline (scan pattern) is required. We analyze the transition patterns and dwell maps to extract such a guideline. First, we define the desirable characteristics of a scan pattern which can help a reader to cover the entire fundus effortlessly and efficiently. Next, we follow a set of rules to extract a recommended pattern with the defined characteristics.

### Optimal transition pattern

**Fig 13:**
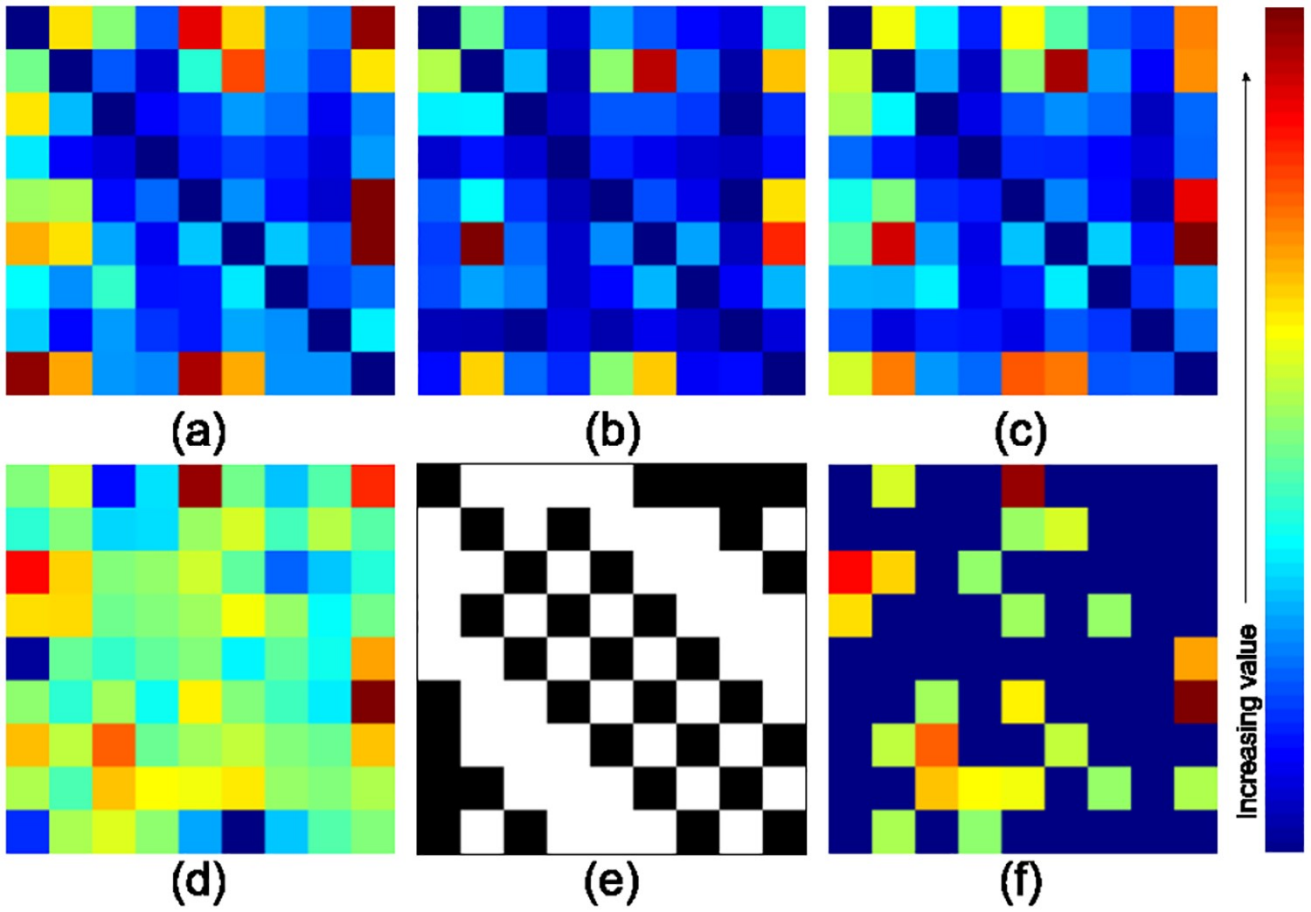
Average transition matrix. Transition matrix (a) for normal cases (b) for abnormal cases (c) average transition matrix *TM*_*avg*_ (d) net transition matrix *TM*_*net*_ (e) binary neighborhood matrix *NM* (e) conditioned net transition matrix *TM*_*c*_.

The analysis was done after *excluding* the responses from novices as the aim is to capture best practice from medically trained subjects. *TM* is calculated for each of these trials and averaged separately into two classes to derive *TM*(*normal*) and *TM*(*abnormal*) as shown in the Fig 13(a) and 13(b). Trials for novices are ignored to avoid false practices.

In order to extract an optimal transition which is applicable for scrutinizing both normal and abnormal images, the transition matrices for these two classes are combined as follows.

$$TM_{avg}=\frac{TM(normal)+TM(abnormal)}{2}$$ (5)

The derived average transition matrix *TM*_*avg*_ is shown in Fig 13(c).

Entries in (*i*, *j*) and (*j*, *i*) positions in *TM*_*avg*_ shows number of transitions from *j* to *i* and from *i* to *j* respectively. These transitions happen due to revisits and should be nullified. Net number of transitions from *j* to *i* is (*i*, *j*) − (*j*, *i*) [22]. Net transition matrix (*TM*_*net*_) is defined as follows.

$$TM_{net}=TM_{avg}-TM_{avg}^{T}$$ (6)

Where, $TM_{avg}^{T}$ is the transpose of *TM*_*avg*_. *TM*_*net*_ is shown in Fig 13(d).

**Fig 14:**
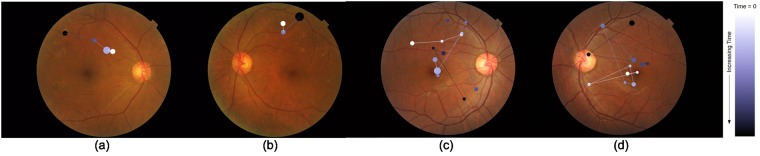
Gaze-pattern of one subject for 4 images. (a), (b) visual search for abnormal cases is terminative (c), (d) visual search for normal cases is exhaustive.

Visual search for an abnormal case is terminative with the search ending as soon as a region with an abnormality is located; whereas for a normal case, search is exhaustive as all the regions are reviewed (see Fig 14). Weak transitions in *TM*_*net*_ might have only occurred during exhaustive search and not during terminative search. Ideally, an optimal transition pattern should progress from strongest to weakest transition to ensure that important transitions happen early on and as soon as an abnormality is located, search can be terminated. Further, an optimal transition pattern should have least number of revisits. In order to maintain easy and smooth eye movements, transitions between neighboring zones are given a higher priority.

In order to satisfy the last condition, a binary neighborhood matrix *NM* is designed. As shown in Fig 13(e), (*i*, *j*) element of *NM* has value equal to 1 if *i*^*th*^ and *j*^*th*^ zones share a common boundary, else the value is 0. *TM*_*net*_ is multiplied with *NM* to allow transitions only between neighboring zones. Also *TM*_*net*_ is anti-symmetric matrix, i.e. (*i*, *j*) = −(*j*, *i*). Duplicate transitions are avoided by setting all elements with negative values to zero. The final constrained matrix *TM*_*c*_ (see Fig 13(f)) is defined as follows.

$$TM_{c}=TM_{net}\circ H\left(TM_{net}\right)\circ NM$$ (7)

Where ∘ signifies Hadamard product and *H*(.) is the Heaviside function.

**Fig 15:**
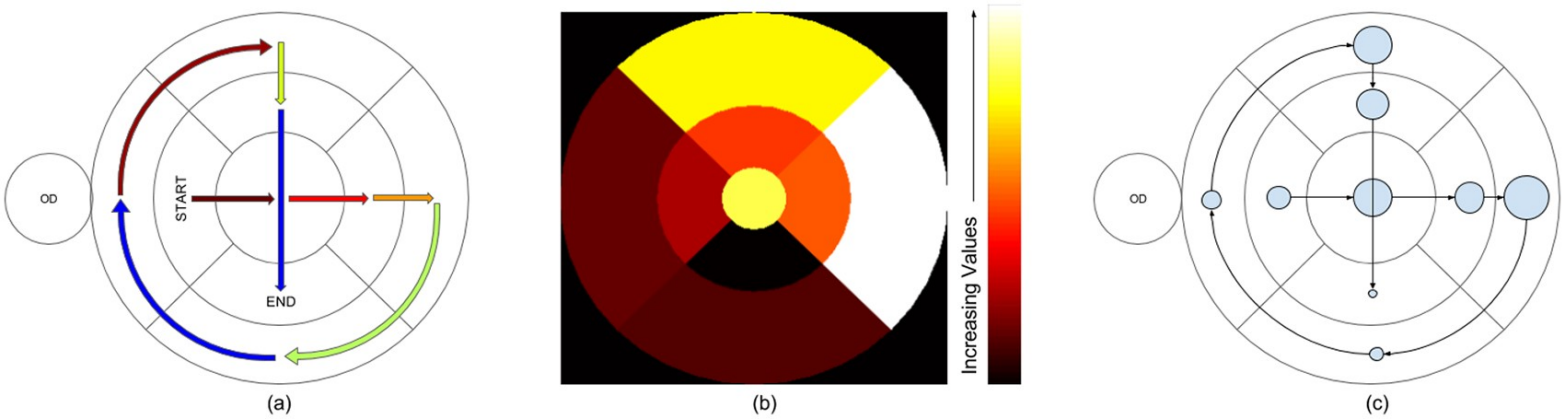
(a) Optimal transition pattern (b) Optimal dwell map (c) Optimal scanning strategy. Transitions in optimal scanning strategy are guided by optimal transition pattern and dwelling by dwell map. The radius of circle represents the amount of required dwelling.

An optimal transition pattern is extracted from *TM*_*c*_ as described next. First, a set of unvisited zones *Z* is defined. In the beginning, all zones are unvisited. Nine zones can be traversed using eight transitions without any revisit. So, arrays *S* and *D* of length = 8 are defined to store the source and destination zones for the transitions respectively. The pair of zones with strongest net transition is initialized as the first source(*S*(0)) and destination(*D*(0)). A search for remaining transition pattern is done such that above stated conditions are satisfied. The destination of a current transition, *D*(*i*), becomes the source of the next transition, *S*(*i* + 1). The next destination is searched from *Z* in three hierarchical levels by looking into *TM*_*c*_. The unvisited zone which has the highest, non-zero net transitions from current source is selected as the destination. Here, only a neighboring zone will be selected as *TM*_*c*_ is constrained with *NM*. If such a zone is not found then the neighboring unvisited zone (which might have zero net transition from current source) with a strong potential destination is selected. If an unvisited neighboring zone is not found then any unvisited zone with strong potential destination is selected. Once a zone is selected as destination, it is removed from set *Z* and appended to set *D*. At the end of 7^*th*^ transition only one zone is left unvisited (length of *Z* = 1), which is selected as the last destination. The final *S* and *D* traces optimal transition pattern. The optimal transition pattern achieved in this manner is shown in Fig 15(a).

### Optimal dwell map

We also aim to derive guidelines on the amount dwell that is preferred for the different zones. The task given to the subjects was to decide the category (normal or abnormal) an image belongs to as soon as possible and hence the visual search for abnormal images is terminative in nature. i.e. once a lesion is located in a certain zone, a decision is given without scrutinizing other zones (see Fig 14(a) and 14(b)). In the absence of lesions, the visual search (the correctly classified) normal cases is more exhaustive in nature (see Fig 14(c) and 14(d)). This is because of the cautiousness exercised to avoid false negatives. It is desirable to derive an optimal dwell map which does not depend on the location of lesions. Hence, *only* trials for normal cases, where medically trained participants registered correct responses, were considered and novices were excluded. The average dwell map is computed as follows.

$$d_{k}=\frac{1}{ij}\sum_{i,j}d_{i,j,k};\;i\in S_{j},j\in N$$ (8)

Where, *S*_*j*_ is a set of medical experts who have given correct response for *j*^*th*^ image. Average dwell map computed using above formula is optimal dwell map and shown in Fig 15(b).

### Optimal scanning strategy

The optimal transition pattern (Fig 15(a)) and optimal dwell map (Fig 15(b)) when combined, describes an optimal scanning strategy (see Fig 15(c)). It is to be noted that dwelling prescribed with optimal dwell map should be considered as relative time spent on various retinal zones. This can be recommended to image readers.

There are several interesting observations that can be made from the derived optimal scanning pattern. Firstly, it can be seen to successfully capture the human preference for the horizontal over vertical and top over bottom directions. Further, transitions are seen to be graceful (not abrupt) which are direct consequences of the conditions imposed to derive this pattern. These two features imply that the optimal scan pattern is efficient, fast and would be easy to follow by any practitioner. It can also be seen that the transitions spiral out, visiting the clinically critical zones near the macula first and then progressing towards peripheral zones. This allows the search to also be terminative in nature.

**Fig 16:**
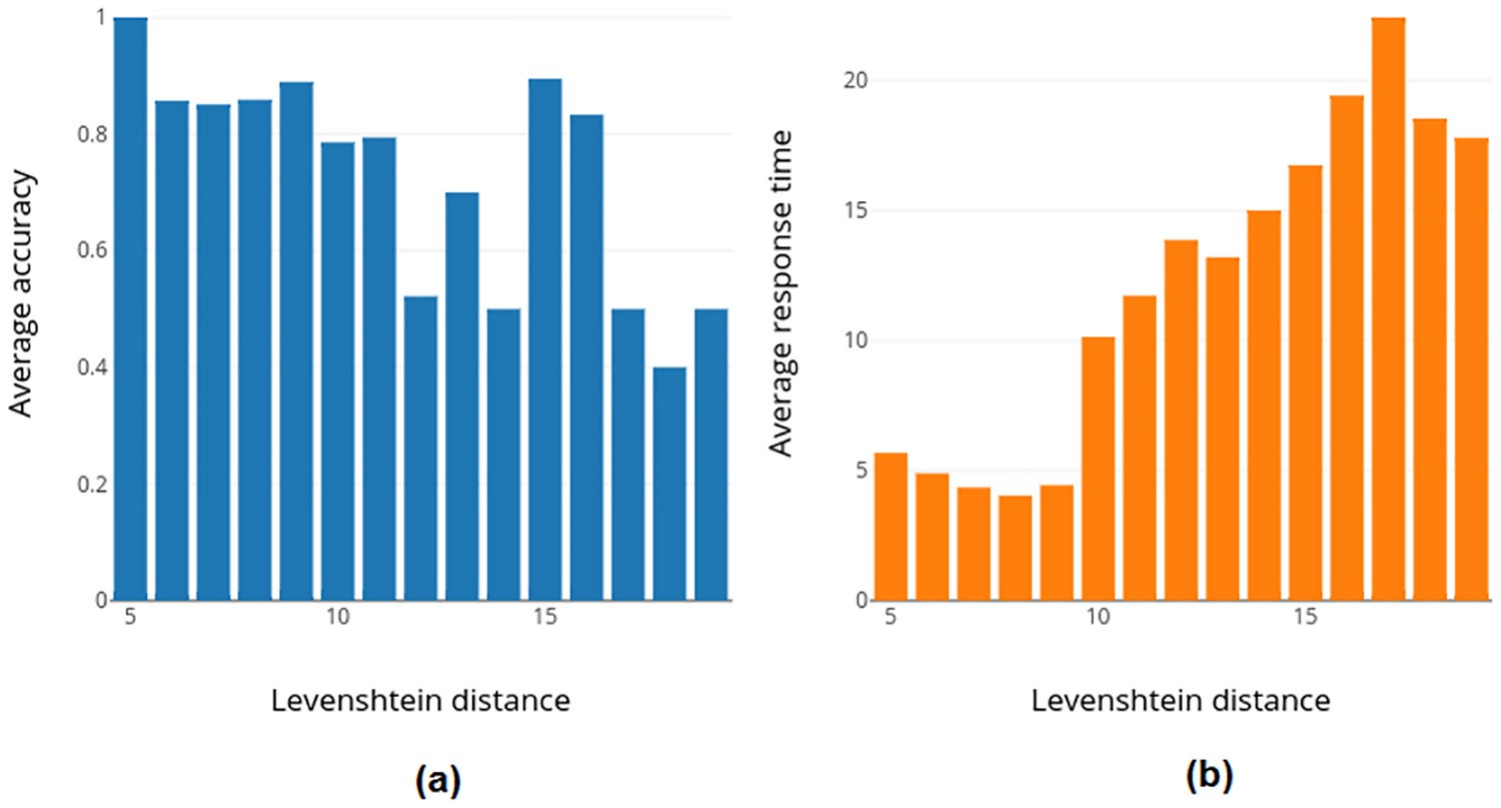
(a) Average accuracy and (b) response time for scanning strategies which are at equal levenshtein distance from optimal strategy.

The above recommended optimal strategy was assessed as follows. The gaze tracks from 2240 responses of all subjects (for 40 images) were quantized into 9 retinal zones (see Fig 10(a)). These quantized gaze tracks represent specific scanning strategies used by a subject. The Levenshtein distance [23] between these scanning strategies and the recommended optimal scanning strategy was calculated. The accuracy and response time for scanning strategies at equal Levenshtein distance from the optimal strategy are averaged and shown in Fig 16. It can be seen that accuracy decreases as a scanning strategy deviates from the optimal strategy (correlation coefficient = -0.7377). Accuracy for strategies which are close to optimal strategy (distance = 5) are higher. Strategies which are close to optimal strategy also have low response time while with increasing deviation, one can observe an increase in response/diagnosis time (correlation coefficient = 0.9351). This establishes the optimality of the proposed strategy also in terms of accuracy and response time.

Finally, based on the optimal dwell map in Fig 15(b), it can be observed that the dwell time is higher (a) in the superior zones than inferior zones and (b) in the temporal zones than nasal zones. Fovea, being a critical part of retinal anatomy, is seen to be scrutinized for a long duration. Dwelling in parafovea zones is less than in perifovea zones. This can be due to two reasons. Firstly, the parafovea zones are proximal to fovea, a dark region. This provides higher contrast for hard exudates enabling these abnormalities to be highly prominent. Thus this region requires less dwell duration. Secondly, parafovea zones are smaller in area than perifovea zones. So the number of fixations in parafovea zones is less; hence total dwell duration is less.

## Conclusions

We conducted an eye tracking study to understand strategies used by various practitioners in DR diagnosis, examine the role of expertise and explore best practices that can be used as a guideline for image readers.

Results indicate that practitioners use mainly two types of strategies, namely, dwelling and tracing. Dwelling is characterized by long fixations, short gaze-tracks and minimum revisits. Whereas, tracing is characterized by short fixations, long gaze-tracks and more revisits. Dwelling, opposed to tracing, was found to lead to accurate and fast diagnosis irrespective of the expertise of a subject.

A recommendation is derived which prescribes (a) the order in which retinal zones should be scrutinized and (b) the required dwelling for each zone. It is possible to use different sets of rules to derive different optimal transition pattern. Recommendation derived using our set of conditions was found to lead to accurate and fast diagnosis.

The key strengths of the reported work are as follows. (a) This is the first attempt to understand visual perception for DR images with eye-tracing techniques. (b) The study participants (56 of them) are from leading eye hospitals, medium and small eye clinics and had various level of experience. This was done to ensure generality of the findings. (c) Analysis of eye-tracking data is done in non-traditional way with newly defined quantities. (d) Scanning strategies and role of expertise on performance are analyzed. (e) A recommendation is derived which can be used to train readers in image reading centers. Limitations of this work are as follows. (a) Study is conducted with 40 images. This is a small number of images compared to actual number of cases a typical reader or expert handles in a day. Results may vary/improve if study is done with more number of images. (b) Study is limited to lesions related to early detection of DR. The optimal scanning strategy may not be effective for advance stages of DR. The future direction includes: (a) field testing of this recommended pattern i.e. to verify that the optimal strategy helps readers in accurate and fast diagnosis. Once this is verified, optimal strategy can be taught to readers under training; (b) designing a CAD solution using the optimal scanning strategy.
